# Supplementary material for: NAAA-regulated lipid signaling in monocytes controls the induction of hyperalgesic priming in mice
Source: Nat Commun. 2024 Feb 24;15:1705. doi: 10.1038/s41467-024-46139-5 (PMC10894261; doi:10.1038/s41467-024-46139-5)
Supplement: Supplementary file 1 — Supplementary Information [file 41467_2024_46139_MOESM1_ESM.pdf]

Supplemental material

NAAA-regulated lipid signaling in monocytes controls the induction of hyperalgesic priming in mice

Yannick Fotio[1], Alex Mabou Tagne[1*], Erica Squire[1*], Hye-lim Lee[1], Connor M. Phillips[2], Kayla Chang[1], Faizy Ahmed[1], Andrew S. Greenberg[3], S. Armando Villalta[2,4], Vanessa M. Scarfone[5], Gilberto Spadoni[6], Marco Mor[7], and Daniele Piomelli[1,8,9,&]

[1]Department of Anatomy and Neurobiology, University of California Irvine, Irvine, CA, USA.
[2]Department of Physiology and Biophysics, University of California, Irvine, CA, USA
[3]Human Nutrition Research Center, Tufts University, Boston, MA, USA
[4]Department of Neurology, University of California, Irvine, CA, USA
[5]Sue and Bill Gross Stem Cell Research Center, University of California Irvine, CA, USA
[6]Dipartimento di Scienze Biomolecolari, Università di Urbino "Carlo Bo," Urbino, Italy.
[7]Dipartimento di Scienze degli Alimenti e del Farmaco, Università di Parma, Parma, Italy.
[8]Department of Biological Chemistry, University of California Irvine, Irvine, CA, USA.
[9]Department of Pharmaceutical Sciences, University of California Irvine, Irvine, CA, USA.

* These authors contributed equally

[&] Corresponding author: Daniele Piomelli (piomelli@hs.uci.edu). Gillespie Neuroscience Res. Facility (room 3101), 837 Health Sciences Rd., Irvine, CA 92697.

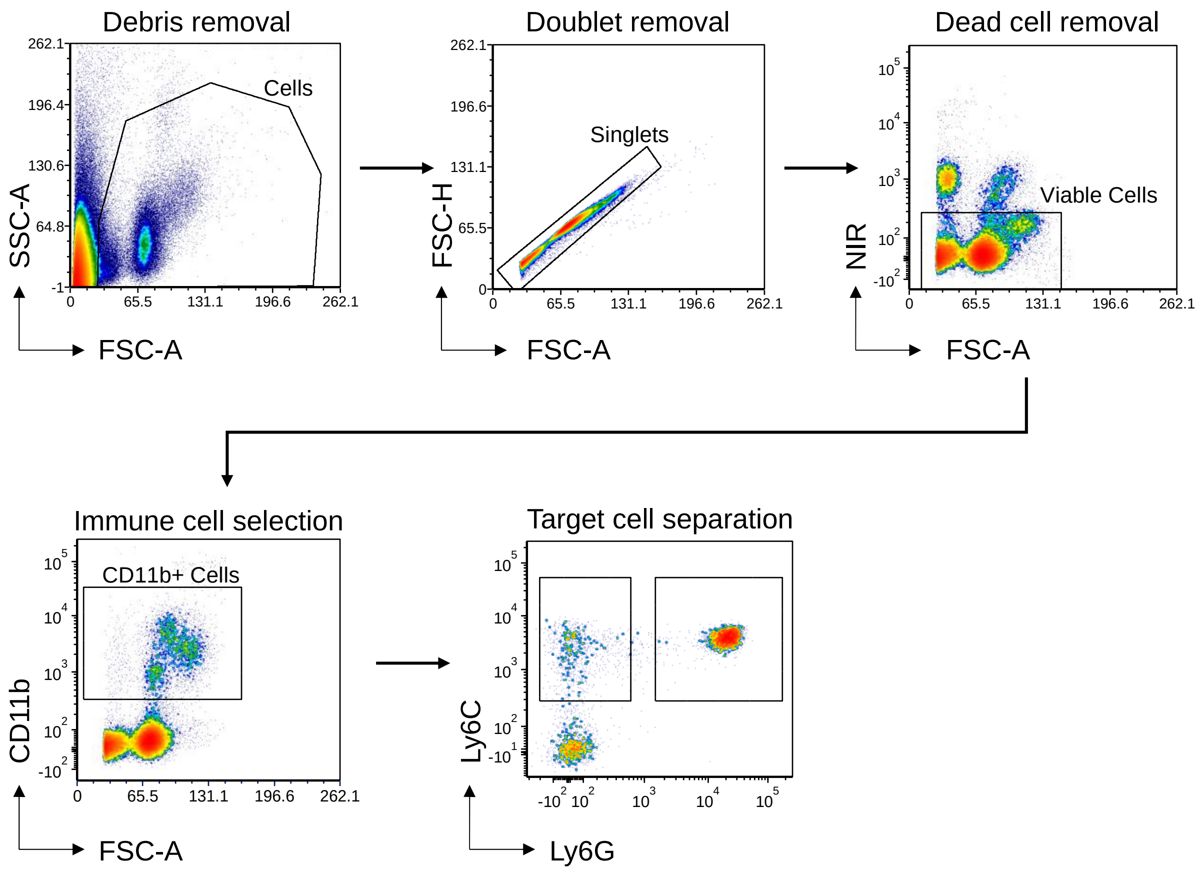

Figure S1: Debris removal and gating strategy for FACS analyses.

Tissue debris was removed by gating on the side scatter area (SSC-A) versus forward scatter area (FSC-A). Cells with a high FSC-A/SSC-A ratio were selected and subjected to forward scatter height (FSC-H) versus FSC-A separation to isolate single cells, which displayed a ratio of ~1. After removal of Zombie NIR^{+} dead cells, viable single cells were isolated based on expression of marker proteins of interest, including CD11b, CD45, Ly6C, and Ly6G. CD11b^{+} Ly6C^{high} Ly6G^{low} cells were categorized as ‘classical’ monocytes, CD11b^{+} Ly6C^{low} Ly6G^{low} cells as non-classical monocytes, CD11b^{+} Ly6C^{high} Ly6G^{high} as neutrophils, and CD11b^{+} CD45^{low} as spinal cord microglia (not shown)[36,37].

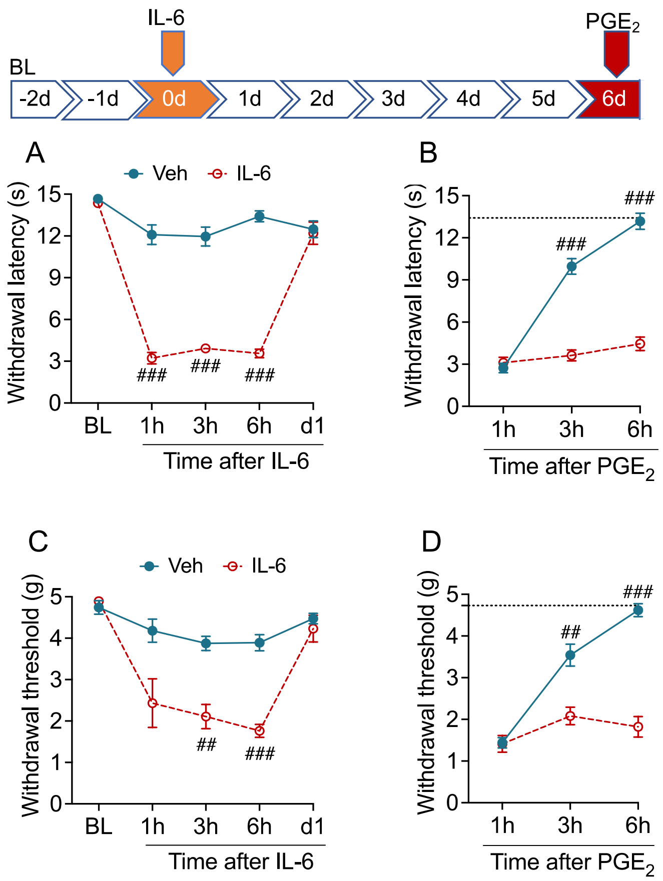

Figure S2: HP model used in the present experiments. Male mice were given intraplantar injections (right hind paw) of a priming agent (e.g., IL-6; n=7) or its vehicle (n=8) on day 0 of the experiment and nociception (**A**: ipsilateral heat hypersensitivity, paw withdrawal latency in seconds**; C**: ipsilateral mechanical hypersensitivity, paw withdrawal threshold in grams) was assessed for the following 24 hours. Six days later, the algogenic prostanoid PGE_2 was administered in the same paw and hypersensitivity (**B**: heat; **D**: mechanical) was measured for the following 6 hours.

Data are expressed as mean ± S.E.M. and were analyzed by two-way repeated measure ANOVA followed by Bonferroni's multiple comparison test. P values versus vehicle are shown. Dotted lines indicate baseline withdrawal latency. Source data are provided as a Source Data file.

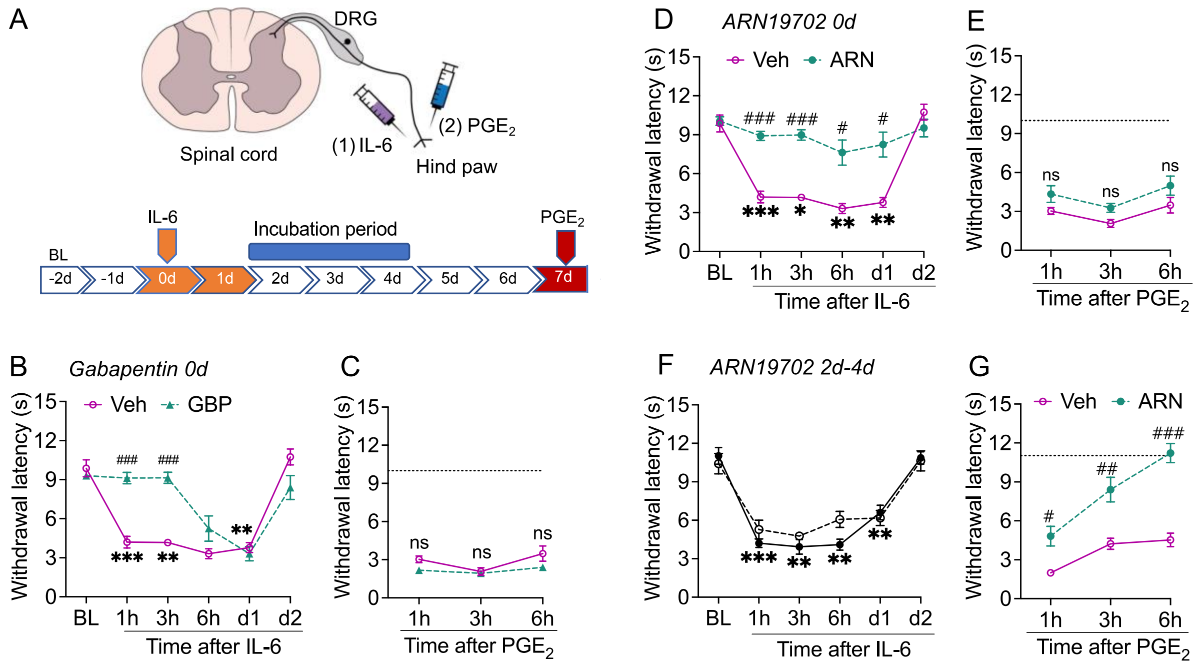

Figure S3: NAAA is required for HP induction in female mice.

(**A**) Protocol used in the present experiments. Priming was induced in female mice by intraplantar administration of IL-6 and was assessed 6 days later by injecting PGE_2 at the same site. Ipsilateral heat hypersensitivity (paw withdrawal latency, s) was measured under baseline (BL) conditions (-2d, -1d) and immediately after administration of IL-6 (0d), PGE_2 (7d), or vehicle (0d and 7d). The blue bar marks the HP incubation period. Compared to males, female mice exhibited a longer response to IL-6 (see **B**, below). For this reason, gabapentin and ARN19702 administrations were conducted on 2d-4d and PGE_2 injection on 7d.

(**B-E**) Heat hypersensitivity after IL-6 injection in female mice (n=6-7 per group) that had received gabapentin (GBP, 50 mg-kg^{-1}; green triangles, **B**), ARN19702 (ARN, 30 mg-kg^{-1}; green circles, **D**), or their vehicle (Veh, magenta circles) 1 h before IL-6. Effect of PGE_2 in mice treated with GBP (**C**, n=6), ARN19702 (**E**, n=7), or their respective vehicles (n=6).

(**F, G**) IL-6-primed mice were treated on 1d-3d with vehicle (n=6) or ARN19702 (30 mg-kg^{-1}; n=9). (**F**) Hypersensitive response to IL-6 before administration of vehicle (open symbols) or ARN19702 (closed symbols). (**G**) Effects of PGE_2 in IL-6-primed mice that had received vehicle (magenta circles) or ARN19702 (green triangles).

Data are presented as mean ± S.E.M. and were analyzed by two-way repeated measure ANOVA followed by Bonferroni's multiple comparison test. $^{***}P < 0.001$, $^{**}P < 0.01$, $^{*}P < 0.05$, versus baseline; $^{\#\#\#}P < 0.001$, $^{\#\#}P < 0.01$, $^{\#}P < 0.05$, versus vehicle or Wt. ns, non-significant.

P values versus vehicle are indicated, when possible. Dotted lines indicate baseline withdrawal latency. Source data are provided as a Source Data file.

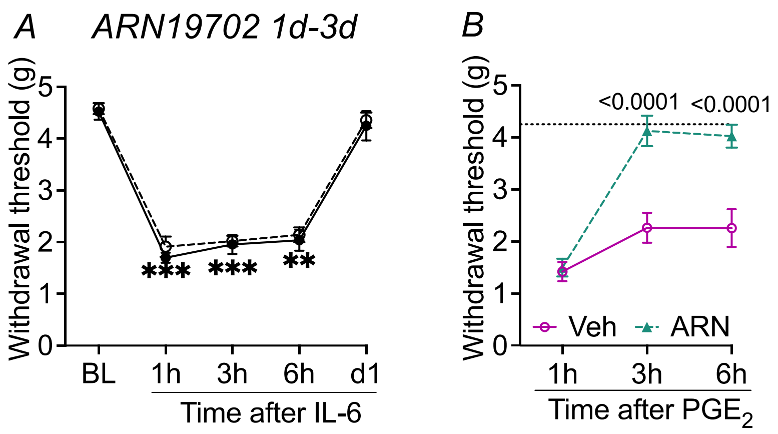

Figure S4: NAAA is required for HP initiation. Effect of ARN19702 on mechanical hypersensitivity.

Priming was induced in male mice by intraplantar administration of IL-6 and was assessed 6 days later by injecting PGE_2 at the same site. Ipsilateral mechanical hypersensitivity (paw withdrawal threshold in grams) was measured under baseline (BL) conditions (-2d, -1d) and immediately after administration of IL-6 (0d), PGE_2 (6d), or vehicle (0d and 6d).

(**A**) Self-resolving mechanical hypersensitivity elicited by IL-6.

(**B**) Effects of PGE_2 on mechanical hypersensitivity in IL-6-primed mice treated with ARN19702 (n=8) or its vehicle (n=7) on day 1-3 following IL-6 challenge.

Data are presented as mean ± S.E.M. and were analyzed by two-way repeated measure ANOVA followed by Bonferroni's multiple comparison test. ***P < 0.001, **P < 0.01, versus baseline. P values versus vehicle are shown, when possible. Dotted lines indicate baseline withdrawal latency. Source data are provided as a Source Data file.

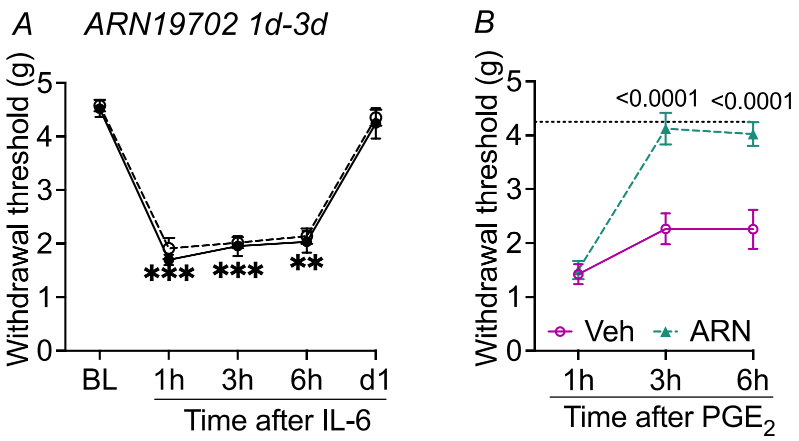

Figure S5: Gabapentin does not prevent HP induction.

(**A**) Response to IL-6 before administration of vehicle (open black circles) or gabapentin (closed black triangles). No differences among groups were observed.

(**B**) Effects of PGE_2 in IL-6-primed mice that had received vehicle (Veh, magenta circles) or gabapentin (GBP, green triangles) on 1d-3d after IL-6.

Data are presented as mean ± S.E.M. and were analyzed by two-way repeated measure ANOVA followed by Bonferroni's multiple comparison test. ***$P < 0.001$, versus baseline (n = 7 mice per group). Dotted line indicates baseline withdrawal latency. Source data are provided as a Source Data file.

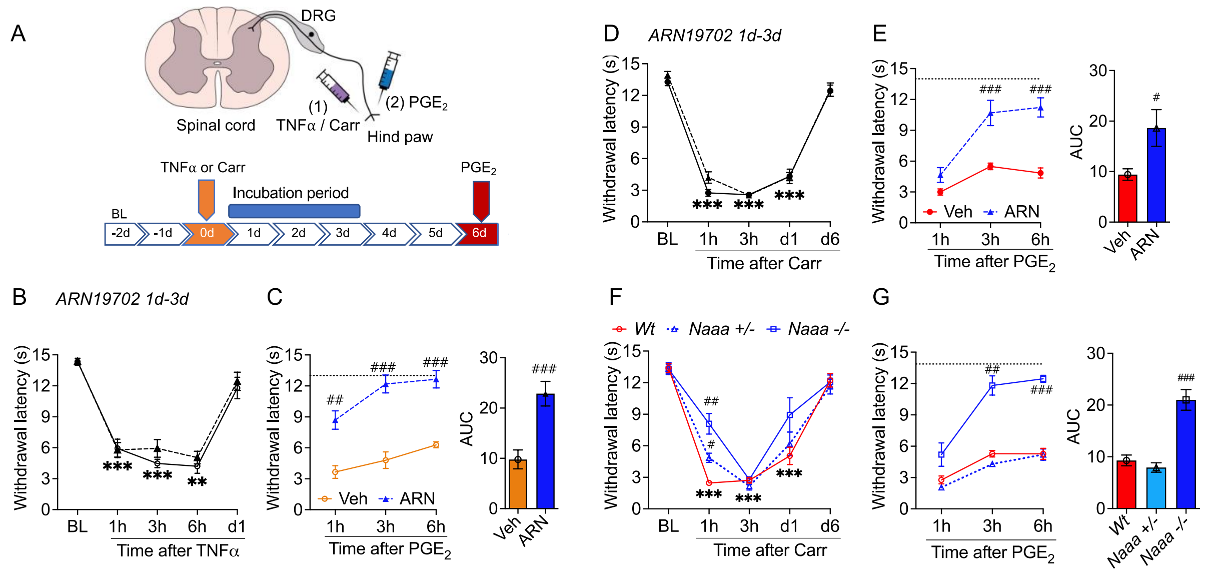

Figure S6: NAAA is required for HP induction by TNFα or carrageenan.

(**A**) Model used in the present experiments. Priming was induced in male mice by intraplantar administration of either TNFα or carrageenan (Carr) and was assessed 6 days later by injecting PGE_2 at the same site. Ipsilateral heat hypersensitivity (paw withdrawal latency, s) was measured under baseline (BL) conditions (-2d, -1d) and immediately after administration of the priming stimulus (0d), PGE_2 (6d), or vehicle (0d and 6d). The blue bar marks the HP incubation period. The illustration was partially generated with BioRender.com

(**B, C**) TNFα-primed mice were treated on 1d-3d with vehicle or ARN19702 (30 mg-kg^{-1}). (**B**) Response to IL-6 before administration of vehicle (open black circles, n=8) or ARN19702 (closed black triangles, n=8). (**C**) Effects of PGE_2 in TNF-α-primed mice that had received vehicle (Veh, orange circles) or ARN19702 (blue triangles). Right panel, area under the curve (AUC).

(**D, E**) Carrageenan-primed mice were treated on 1d-3d with vehicle or ARN19702 (30 mg-kg^{-1}). (**D**) Response to IL-6 before administration of vehicle (closed black circles, n=10) or ARN19702 (closed black triangles, n=12). (**E**) Effects of PGE_2 in carrageenan-primed mice that had received vehicle (red circles) or ARN19702 (blue triangles).

(**F**) Heat hypersensitivity after carrageenan injection in homozygous *Naaa*$^{-/-}$ mice (blue triangles, n=7), heterozygous *Naaa*$^{+/-}$ mice (blue squares, n=8), and their wild-type littermates (red circles, n=7). (**G**) Effects of PGE_2 in the same animals. Right panel, AUC.

Data are presented as mean ± S.E.M. and were analyzed by two-tailed unpaired Student's *t* test (AUC) or two-way repeated measure ANOVA followed by Bonferroni's multiple comparison test. ***P < 0.001, **P < 0.01, versus baseline; $^{\#\#}$P < 0.01, $^{\#}$P < 0.05, versus Wt.

P values versus *Wt* or vehicle are shown, when possible. Dotted lines indicate baseline withdrawal latency. Source data are provided as a Source Data file.

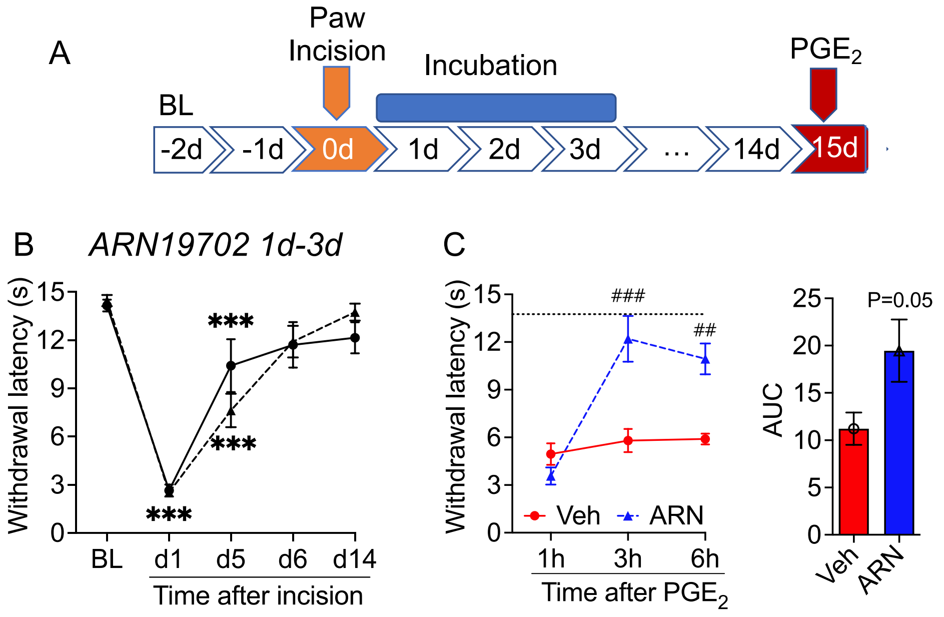

Figure S7: NAAA is required for HP induction by paw incision.

(**A**) Model used in the present experiments. Priming was induced in male mice with a surgical incision of the plantar aspect of the right hind paw and was assessed 15 days later by injecting PGE_2 at the same site. Ipsilateral heat hypersensitivity (paw withdrawal latency, s) was measured under baseline (BL) conditions (-2d), immediately after administration of the priming stimulus (0d) or PGE_2 (15d). The blue bar marks the HP incubation period during which mice received ARN19702 (30 mg-Kg^{-1}) or its vehicle.

(**B**) Nociceptive response after the incision and before administration of vehicle (closed black circles, n=7) or ARN19702 (closed black triangles, n=8).

(**C**) Effects of PGE_2 in incision-primed mice that had received vehicle (Veh, red circles) or ARN19702 (blue triangles). Right panel, area under the curve (AUC).

Data are presented as mean ± S.E.M. and were analyzed by two-way repeated measure ANOVA followed by Bonferroni's multiple comparison test or two-tailed unpaired Student's *t* test (AUC). P values versus baseline (BL) or vehicle are shown. Dotted lines indicate baseline withdrawal latency. Source data are provided as a Source Data file.

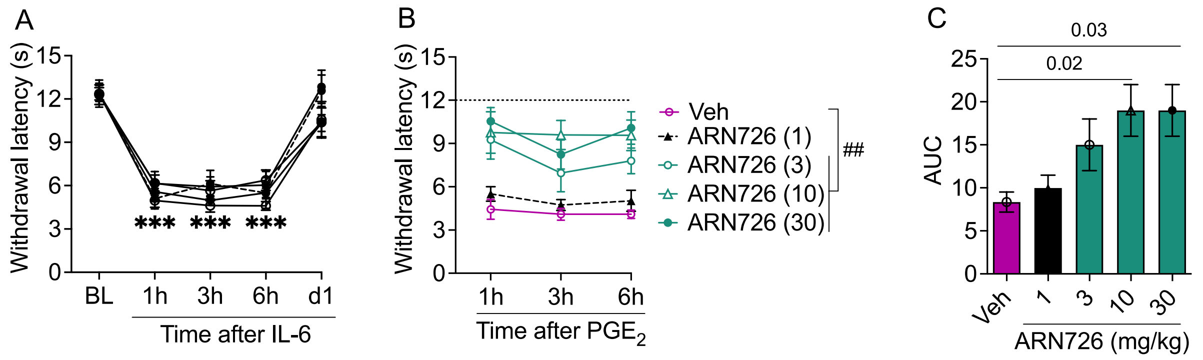

Figure S8: Systemic ARN726 administration prevents HP induction.

(**A**) Response to IL-6 before administration (black symbols) of vehicle or ARN726. No differences among groups were observed.

(**B**) Effects of PGE_2 injection in IL-6-primed mice treated (IP) with vehicle (magenta circles, n=6) or ARN726 at the following doses: 1 mg-kg^{-1} (black triangles, n=8); 3 mg-kg^{-1} (open green circles, n=8); 10 mg-kg^{-1} (green triangles, n=8); and 30 mg-kg^{-1} (closed green circles, n=7).

(**C**) Area Under the Curve (AUC) of the effects of PGE_2 in primed animals.

Data are shown as mean ± S.E.M. and were analyzed by one-way (AUC) or two-way repeated measure ANOVA, followed by Dunnett's or Bonferroni's multiple comparison test. ***P < 0.001, versus baseline (BL); $^{\#\#}$P < 0.01 versus vehicle. P values versus vehicle are shown, when possible. Dotted line indicates baseline withdrawal latency. Source data are provided as a Source Data file.

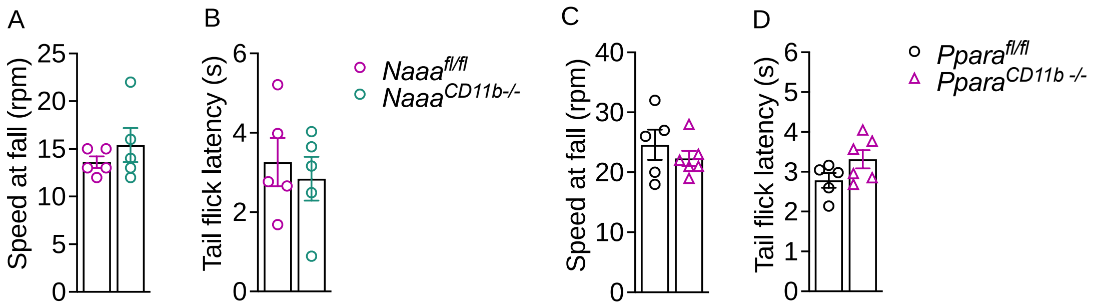

Figure S9: Motor coordination and baseline nociceptive thresholds in CD11b$^+$ cell-selective *Naaa*$^{-/-}$ and *Ppara*$^{-/-}$ mice.

(**A, C**) No differences in motor coordination were seen across mouse lines. *Naaa*$^{fl/fl}$ and *Ppara*$^{fl/fl}$, animals lacking *Naaa* or *Ppara* in CD11b$^+$ cells did not show a difference in the speed at fall [in rotation per min (rpm)] in the accelerated rotarod test.

(**B, D**) No differences were observed in baseline nociceptive thresholds across mouse lines. *Naaa*$^{fl/fl}$, magenta circles; *Ppara*$^{fl/fl}$, open black circles; *Naaa*$^{CD11b-/-}$, green circles; *Ppara*$^{CD11b-/-}$, magenta triangles.

Data are shown as mean ± S.E.M. and were analyzed using Student's *t* test (n = 5). Source data are provided as a Source Data file.

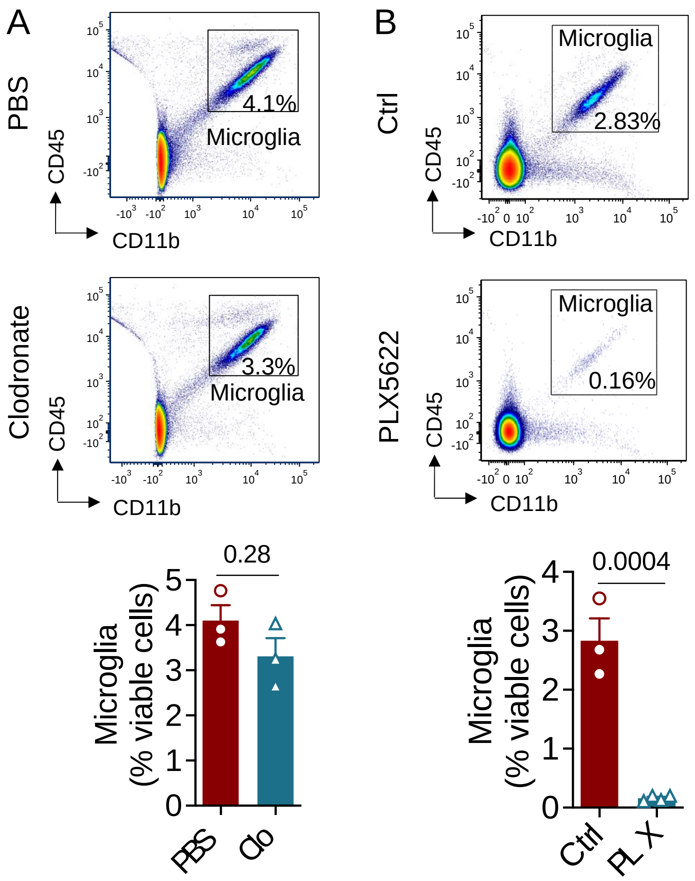

Figure S10: FACS analysis of spinal cord microglia.

(**A**) Gating strategy for isolation and quantification of spinal cord microglia from PBS- and clodronate-treated mice. *Top*, CD11b^{+}CD45^{low} populations of microglia from a mouse after PBS treatment (≈4.1%). *Bottom*: clodronate treatment did not significantly alter the number of microglia (≈3.3%).

(**B**) Gating strategy for isolation and quantification of spinal cord microglia from mice fed chow containing vehicle (Ctrl) or PLX5622 (PLX) for 20 days. *Top*, population of CD11b^{+}CD45^{low} microglia in the spinal cord of a Ctrl mouse. *Bottom*, PLX depleted microglia by approximately 94%. Data are representative of three biological replicates. They are shown as mean ± S.E.M. and were analyzed by unpaired two-tailed Student's *t* test. P value versus Ctrl is indicated. Source data are provided as a Source Data file.

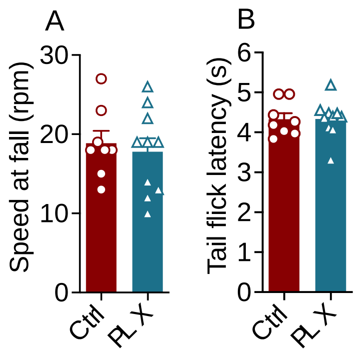

Figure S11: Motor coordination and baseline nociceptive thresholds in mice treated with CSF-1 receptor antagonist PLX5622.

(**A**) No differences in motor coordination were observed across groups. Mice fed chow containing vehicle (Ctrl, red bars, n=8) or PLX5622 (PLX, blue bars, n=10) for 20 days did not show a difference in the speed at fall [in rotation per min (rpm)] in the accelerated the rotarod test.

(**B**) Baseline nociceptive thresholds. PLX-treated mice and their control (Ctrl) counterparts did not exhibit any difference in tail flick latency (s) during the tail immersion test.

Data are shown as mean ± S.E.M. and were analyzed using two-tailed unpaired Student's *t* test. Source data are provided as a Source Data file.

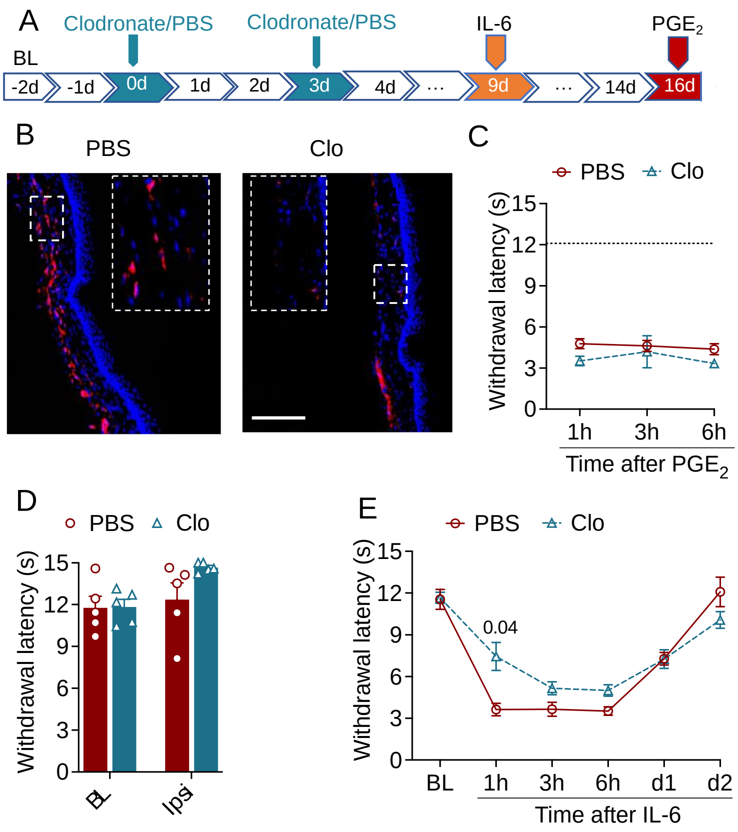

Figure S12: Effects of intraplantar clodronate injection on HP induction.

(**A**) Protocol used in the present experiments. Liposomes containing clodronate (Clo) or vehicle (PBS) were injected in the mouse right hind paw on day 0 and again on day 3 of the experiment. IL-6 and PGE_2 were administered in the same paw six days and 13 days later, respectively.

(**B**) Immunofluorescent images showing CD68+ cell bodies (red) and DAPI-stained nuclei (blue) in paws treated with PBS (left) or clodronate (right) six days earlier. Magnification, x10. Hatched squares show highlighted areas at higher magnification (x40). Scale bar, 200 µm. Results were obtained in three independent biological replicates. Representative images are shown here.

(**C**) Nociceptive responses (heat hypersensitivity, withdrawal latency, s) to intraplantar PGE_2 injection in paws treated with PBS (red circles, n=6) or clodronate liposomes (blue triangles, n=7).

(**D**) Baseline nociceptive responses (paw withdrawal latency, s) in paws treated with PBS (red circles/bars, n=5) or clodronate liposomes (blue triangles/bars, n=5).

(**E**) Nociceptive responses (heat hypersensitivity) to intraplantar IL-6 injection in paws treated with PBS (red circles, n=6) or clodronate liposomes (blue triangles, n=7).

Data are shown as mean ± S.E.M. and were analyzed by multiple unpaired two-tailed *t* test or two-way repeated measure ANOVA, followed by Bonferroni's multiple comparison. P value

versus PBS is shown. Dotted line indicates baseline withdrawal latency. Source data are provided as a Source Data file.

Name	Fluorophore	Source	Clone	Dilution	Cat #
Anti-CD45 antibody	PE-Cyanine7	Invitrogen	30-F11	1:50	25-0451-82
Anti-CD11b antibody	Brilliant Violet 421	Biolegend	M1/70	1:50	101236
Anti-Ly6C antibody	APC	Biolegend	HK1.4	1:50	128016
Anti-Ly6G antibody	PE	Biolegend	1A8	1:50	127608
Zombie NIR	APC-Cy7	Biolegend	N/A	1:100	423106

Table S1: Reagents used for FACS analysis.

PK parameters of ARN726	
C_{max} (ng-mL^{-1})	333.82
T_{max} (min)	15
K_{el} (h^{-1})	2.9
$t_{1/2}$ (min)	15
AUC_{plasma} (h x ng-mL^{-1})	63.33

Table S2: Pharmacokinetic profile of ARN726 in mice.

ARN726 was administered to male mice (10 mg-kg^{-1}, IP). C_{max}, maximal plasma concentration; T_{max}, time at which C_{max} is reached; K_{el}, elimination rate constant; $t_{1/2}$, half-life of elimination; AUC, area under the curve. Data are the average of 5 biological replicates.

Target	Antibody Clone	Supplier	Metal Mass	Element	Concentration (ug/ml)
CD44	IM7	Biolegend	106	Cd	1
CD14	Sa14-2	Biolegend	110	Cd	4
CD39	24DMS1	Biolegend	111	Cd	4
CD64	X54-5/7.1	Biolegend	113	Cd	4
IFNg	XMG1.2	Biolegend	114	Cd	1
CD45	30-F11	Biolegend	116	Cd	1
Ly_6G	1A8	Biolegend	141	Pr	2
Ly-6C/G	RB6-8C5	Biolegend	142	Nd	2

MerTK	DS5MMER	Invitrogen	144	Nd	4
F4/80	BM8	Biolegend	146	Nd	4
TNFa	MP6-XT22	Biolegend	147	Sm	1
Ly_6C	HK.14	Biolegend	150	Nd	2
CD62L	HK.14	Biolegend	151	Eu	4
CD73	TY/11.8	Biolegend	152	Sm	4
CD43	Polyclonal	Invitrogen	153	Eu	4
CD38	90	Biolegend	154	Sm	4
CD11a	M17/4	Biolegend	155	Gd	2
CD68	FA-11	Biolegend	156	Gd	1
IL-17A	TC11-18H10.1	Biolegend	159	Tb	1
pCREB	E113	Abcam	160	Gd	1
IL-6	MP5-20F3	Biolegend	161	Dy	1
CD163	Polyclonal	Bioss	163	Dy	4
CD161 NK1.1	PK136	Biolegend	164	Dy	2
CX3CR1	polyclonal	R&D Systems	166	Er	4
CD115 (CSF1R)	AFS98	Invitrogen	167	Er	4
PPARa	Polyclonal	Bioss	168	Er	1
CD206	C068C2	Fluidigm/SBT	169	Tm	4
Iba1	Polyclonal	Wako	170	Er	1
Mac2_Gal3	M3/38	Cedarlane	171	Yb	4
CD11b	M1/70	Biolegend	172	Yb	2
CCR2	Polyclonal	Invitrogen	174	Yb	4
I-A/I-E (MHCII)	MS/114.15.2	Fluidigm/SBT	175	Lu	2
CD11c	N418	Fluidigm/SBT	209	Bi	2

Table S3: Reagents used for CyTOF.
