# Supplementary material for: NAAA-regulated lipid signaling in monocytes controls the induction of hyperalgesic priming in mice
Source: Nat Commun. 2024 Feb 24;15:1705. doi: 10.1038/s41467-024-46139-5 (PMC10894261; doi:10.1038/s41467-024-46139-5)
Supplement: Supplementary file 3 — Reporting Summary [file 41467_2024_46139_MOESM3_ESM.pdf]

nature portfolio

Corresponding author(s): Prof. Daniele Piomelli

Last updated by author(s): January 30, 2023

Reporting Summary

Nature Portfolio wishes to improve the reproducibility of the work that we publish. This form provides structure for consistency and transparency in reporting. For further information on Nature Portfolio policies, see our Editorial Policies and the Editorial Policy Checklist.

Statistics

For all statistical analyses, confirm that the following items are present in the figure legend, table legend, main text, or Methods section.

n/a	Confirmed	
☐	☒	The exact sample size (*n*) for each experimental group/condition, given as a discrete number and unit of measurement
☐	☒	A statement on whether measurements were taken from distinct samples or whether the same sample was measured repeatedly
☐	☒	The statistical test(s) used AND whether they are one- or two-sided<br>*Only common tests should be described solely by name; describe more complex techniques in the Methods section.*
☒	☐	A description of all covariates tested
☐	☒	A description of any assumptions or corrections, such as tests of normality and adjustment for multiple comparisons
☐	☒	A full description of the statistical parameters including central tendency (e.g. means) or other basic estimates (e.g. regression coefficient) AND variation (e.g. standard deviation) or associated estimates of uncertainty (e.g. confidence intervals)
☐	☒	For null hypothesis testing, the test statistic (e.g. *F*, *t*, *r*) with confidence intervals, effect sizes, degrees of freedom and *P* value noted<br>*Give P values as exact values whenever suitable.*
☒	☐	For Bayesian analysis, information on the choice of priors and Markov chain Monte Carlo settings
☒	☐	For hierarchical and complex designs, identification of the appropriate level for tests and full reporting of outcomes
☒	☐	Estimates of effect sizes (e.g. Cohen's *d*, Pearson's *r*), indicating how they were calculated

Our web collection on statistics for biologists contains articles on many of the points above.

Software and code

Policy information about availability of computer code

Data collection	MassHunter Software version B.08.00 (for LC/MS-MS analyses), FCS Express version 7.18.0025. (for cell sorting and FACS), CyTOF Software version 7.0.8493® (for high dimension mass cytometry), Keyence BZ-X Analyzer software (for fluorescence image acquisition)
Data analysis	All statistical analyses were performed with Graphpad prism version 9.1

For manuscripts utilizing custom algorithms or software that are central to the research but not yet described in published literature, software must be made available to editors and reviewers. We strongly encourage code deposition in a community repository (e.g. GitHub). See the Nature Portfolio guidelines for submitting code & software for further information.

Data

Policy information about availability of data

All manuscripts must include a data availability statement. This statement should provide the following information, where applicable:

- Accession codes, unique identifiers, or web links for publicly available datasets
- A description of any restrictions on data availability
- For clinical datasets or third party data, please ensure that the statement adheres to our policy

All data generated in this study are readily available within the paper and its supplementary files. Source data are provided with this paper.

Research involving human participants, their data, or biological material

Policy information about studies with human participants or human data. See also policy information about sex, gender (identity/presentation), and sexual orientation and race, ethnicity and racism.

Reporting on sex and gender	n/a
Reporting on race, ethnicity, or other socially relevant groupings	n/a
Population characteristics	n/a
Recruitment	n/a
Ethics oversight	n/a

Note that full information on the approval of the study protocol must also be provided in the manuscript.

Field-specific reporting

Please select the one below that is the best fit for your research. If you are not sure, read the appropriate sections before making your selection.

☒ Life sciences ☐ Behavioural & social sciences ☐ Ecological, evolutionary & environmental sciences

For a reference copy of the document with all sections, see nature.com/documents/nr-reporting-summary-flat.pdf

Life sciences study design

All studies must disclose on these points even when the disclosure is negative.

Sample size	Sample size for behavioral studies was predetermined by power analysis (alpha = 0.05, 1-beta = 0.8, effect size ~35%, n = 7 per group). For molecular and biochemical studies, sample size (n=4-6 animals per group) was based on prior experience with the proposed models.
Data exclusions	No treatment sample was excluded
Replication	To ensure replication, all RT-qPCR and biochemical data were repeated in at least 4 mice per group per time point.<br>For in vivo assays, a minimum of 8 biological replicates were used. Replication information in included in the figure legend.
Randomization	All samples were randomly allocated to treatment groups.
Blinding	Data acquisition and analysis were performed under blinded conditions.

Reporting for specific materials, systems and methods

We require information from authors about some types of materials, experimental systems and methods used in many studies. Here, indicate whether each material, system or method listed is relevant to your study. If you are not sure if a list item applies to your research, read the appropriate section before selecting a response.

Materials & experimental systems

n/a	Involved in the study	
☐	☒	Antibodies
☒	☐	Eukaryotic cell lines
☒	☐	Palaeontology and archaeology
☐	☒	Animals and other organisms
☒	☐	Clinical data
☒	☐	Dual use research of concern
☒	☐	Plants

Methods

n/a	Involved in the study	
☒	☐	ChIP-seq
☐	☒	Flow cytometry
☒	☐	MRI-based neuroimaging

Antibodies

Antibodies used	1. For FACS (Name, Fluorophore, Source, Clone, Dilution, Cat #):<br><br>Anti-CD45, PE-Cyanine7, Invitrogen, 30-F11, 1:50, 25-0451-82

Anti-CD11b, Brilliant Violet 421, Biolegend, M1/70, 1:50, 101236
Anti-Ly6C, APC, Biolegend, HK1.4, 1:50, 128016
Anti-Ly6G, PE, Biolegend, 1A8, 1:50, 127608
Zombie NIR, APC-Cy7, Biolegend, N/A, 1:100 ,423106

2. For IHC: Name (Source, Clone, Dilution, Cat #):
Anti-CD68 (Serotec, FA-11, 1:200, MCA1957); anti-rat Alexa Fluor 594 donkey (Invitrogen, #A21209, 1:200)

3. For CyTOF (Target, Clone, Supplier, Metal Mass, Element, Concentration (ug/ml)):

CD44, IM7, Biolegend, 106, Cd, 1
CD14, Sa14-2, Biolegend, 110, Cd, 4
CD39, 24DMS1, Biolegend, 111, Cd, 4
CD64, X54-5/7.1, Biolegend, 113, Cd, 4
IFNg, XMG1.2, Biolegend, 114, Cd, 1
CD45, 30-F11, Biolegend, 116, Cd, 1
Ly_6G, 1A8, Biolegend, 141, Pr, 2
Ly-6C/G, RB6-8C5, Biolegend, 142, Nd, 2
MerTK, DS5MMER, Invitrogen, 144, Nd, 4
F4/80, BM8, Biolegend, 146, Nd, 4
TNFa, MP6-XT22, Biolegend, 147, Sm, 1
Ly_6C, HK.14, Biolegend, 150, Nd, 2
CD62L, HK.14, Biolegend, 151, Eu, 4
CD73, TY/11.8, Biolegend, 152, Sm, 4
CD43, Polyclonal, Invitrogen, 153, Eu, 4
CD38, 90, Biolegend, 154, Sm, 4
CD11a, M17/4, Biolegend, 155, Gd, 2
CD68, FA-11, Biolegend, 156, Gd, 1
IL-17A, TC11-18H10.1, Biolegend, 159, Tb, 1
pCREB, E113, Abcam, 160, Gd, 1
IL-6, MP5-20F3, Biolegend, 161, Dy, 1
CD163, Polyclonal, Bioss, 163, Dy, 4
CD161, NK1.1 PK136, Biolegend, 164, Dy, 2
CX3CR1, polyclonal, R&D, Systems, 166, Er, 4
CD115 (CSF1R), AFS98, Invitrogen, 167, Er, 4
PPARa, Polyclonal Bioss, 168, Er, 1
CD206, C068C2, Fluidigm/SBT, 169, Tm, 4
Iba1, Polyclonal, Wako, 170, Er, 1
Mac2_Gal3, M3/38, Cedarlane, 171, Yb, 4
CD11b, M1/70, Biolegend, 172, Yb, 2
CCR2, Polyclonal, Invitrogen, 174, Yb, 4
I-A/I-E (MHCII), MS/114.15.2, Fluidigm/SBT, 175, Lu, 2
CD11c, N418, Fluidigm/SBT, 209, Bi, 2

Additional information is available on manufacturers' website.

Validation

All antibodies are commercially available and were validated by the manufacturer for FACS, CyTOF or Immunofluorescence application in mice. See the corresponding manufacturer datasheets or product details section on the webpages for reference, verified reactivity species and validation. For example, The 30-F11 monoclonal antibody reacts with all isoforms of mouse CD45 (ref: Invitrogen's webpage); antiCD45: verified reactivity-->mouse (ref: Biolegend's webpage). Moreover, several labs including ours have further validated these antibodies for used in mice. Please see for reference PMIDs 36626234, 30606613, 35750512, 12480978, 36035791. In addition, for CyTOF analyses, antibodies were further validated in C57BL/6J mice. See Figure 6 of the present article.

Animals and other research organisms

Policy information about studies involving animals; ARRIVE guidelines recommended for reporting animal research, and Sex and Gender in Research

Laboratory animals

We used male and female C57BL/6J mice (22-30 g, 10-12 weeks old) from Jackson Laboratory (Cat #000664). All animals were housed in the animal facility of the University of California Irvine, using standard ventilated clear plastic cages (3-5 per cage) and conventional wood chips bedding. Food and water were available ad libitum. The mice were maintained in a pathogen free-environment on a 12-hr light/dark cycle at controlled temperature (22°C) and humidity (50-60%). Naaa-/-, NaaaCD11b-/-, NaaaCD11b+, PparaCD11b-/- and their wild-type littermates were bred in the lab.

Wild animals

This study did not involve wild animals

Reporting on sex

The present findings largely apply to male and female mice. See Figure S3 for female data

Field-collected samples

This study did not involve samples collected in the field

Ethics oversight

All experimental procedures complied with the ethical regulations for the care and use of laboratory animals promulgated by the National Institutes of Health (NIH) and the International Association for the Study of Pain (IASP). Formal approval was obtained from

the Animal Care and Use Committee of the University of California, Irvine. All possible efforts were made to minimize the number of animals used and their discomfort.

Note that full information on the approval of the study protocol must also be provided in the manuscript.

Plants

Seed stocks	n/a
Novel plant genotypes	n/a
Authentication	n/a

Flow Cytometry

Plots

Confirm that:

☒ The axis labels state the marker and fluorochrome used (e.g. CD4-FITC).

☒ The axis scales are clearly visible. Include numbers along axes only for bottom left plot of group (a 'group' is an analysis of identical markers).

☒ All plots are contour plots with outliers or pseudocolor plots.

☒ A numerical value for number of cells or percentage (with statistics) is provided.

Methodology

Sample preparation	1. Blood was drawn via cardiac puncture and incubated with ammonium-chloride-potassium (ACK) buffer for erythrocyte lysis. Leukocytes were stained with anti-CD11b, Zombie NIR, anti-Ly6C, and anti-Ly6G antibodies in antibody-staining buffer (0.2% BSA and 0.1% sodium azide in PBS) for 30 min at 4ºC.<br>2. The spinal cord underwent enzymatic digestion and mechanical dissociation using a brain dissociation kit (Miltenyi Biotec, Bergisch Gladbach, Germany), following the manufacturer's instructions. Subsequently, the dissociated spinal cord cells were stained with anti-CD11b and anti-CD45 antibodies in antibody-staining buffer (0.2% BSA and 0.1% sodium azide in PBS) for 30 min at 4ºC.
Instrument	BD Arial II Cell Sorter and BD Fusion Cell Sorter
Software	FCS Express 7 (De Novo Software, Pasadena, CA)
Cell population abundance	Viable cell population was collected by FACS and quantified using FCS Express 7. CD11b+ Ly6Chigh Ly6Glow were categorized as classical/inflammatory monocytes (5-11% of viable blood cells); CD11b+ Ly6Clow Ly6Glow were "non-classical" monocytes (~4% of viable blood cells), while neutrophils were defined as CD11b+ Ly6Chigh Ly6Ghig (6-20% of viable blood cells).
Gating strategy	All FACS analysis included the following steps. Tissue debris was removed by gating on the side scatter area (SSC-A) versus the forward scatter area (FSC-A). Cells with a high FSC-A/SSC-A ratio were selected and subjected to to forward scatter height (FSC-H) versus FSC-A analysis to isolate single cells that displayed a ratio of ~1. After removal of Zombie NIR dead cells, viable single cells were removed based on expression of marker protein CD11b, CD45, Ly6C and Ly6G.

☒ Tick this box to confirm that a figure exemplifying the gating strategy is provided in the Supplementary Information.
